# Supplementary material for: Fecal microbiota transplantation from protozoa-exposed donors downregulates immune response in a germ-free mouse model, its role in immune response and physiology of the intestine
Source: PLoS One. 2024 Oct 28;19(10):e0312775. doi: 10.1371/journal.pone.0312775 (PMC11515975; doi:10.1371/journal.pone.0312775)
Supplement: S2 Fig — (PDF) [file pone.0312775.s002.pdf]

S2. The most abundant orders of bacteria in the parasitized and non-parasitized groups of volunteers.

1		Abundance of bacteriome in the B:Onon-parasitized group							Abundance of bacteriome in the non-parasitized group						
2	Consensus_Lineage	P1	P2	P3	P4	P5	P6	P7	NP1	NP2	NP3	NP4	NP5	NP6	NP7
3	Bacteria;Bacteroidetes;Bacteroidia;Bacteroidales;Prevotellaceae;Prevotella;copri	22515	1474	9330	349	1076	10907	33	14553	6664	1047	4090	105	168	101
4	Bacteria;Bacteroidetes;Bacteroidia;Bacteroidales;Prevotellaceae;Prevotella;copri	14033	43	90	15	81	1784	3	5980	528	14	254	1	23	5
5	Bacteria;Firmicutes;Clostridia;Clostridiales;Lachnospiraceae;unclassified;unclassified	8542	1213	442	1389	514	4014	29	1598	43	2173	7470	164	467	43
6	Bacteria;Actinobacteria;Actinobacteria;Bifidobacteriales;Bifidobacteriaceae;Bifidobacterium;unclassified	6634	1331	69	4237	203	9571	96	7472	100	778	3717	17034	43934	155
7	Bacteria;Firmicutes;Clostridia;Clostridiales;Ruminococcaceae;Ruminococcus;bromii	4850	3153	318	13971	2901	2068	49	238	68	4153	3231	67	24	60
8	Bacteria;Actinobacteria;Actinobacteria;Bifidobacteriales;Bifidobacteriaceae;Bifidobacterium;longum	3379	621	402	13530	176	305	58	1586	819	44	16660	19614	2571	151862
9	Bacteria;Bacteroidetes;Bacteroidia;Bacteroidales;Bacteroidaceae;Bacteroides;unclassified	3351	51	18	179	51	613	0	42	96	7	169	83	76	4
10	Bacteria;Firmicutes;Clostridia;Clostridiales;Ruminococcaceae;Oscillospira;unclassified	3048	2085	492	2221	2518	253	40	250	253	2153	219	232	51	64
11	Bacteria;Bacteroidetes;Bacteroidia;Bacteroidales;Bacteroidaceae;Bacteroides;unclassified	2617	34	22	198	12	551	7	108	2	15	153	501	23	1
12	Bacteria;Bacteroidetes;Bacteroidia;Bacteroidales;Prevotellaceae;Prevotella;unclassified	2432	3	1216	9	2	0	4	3621	363	159	2	14	3	2
13	Bacteria;unclassified;unclassified;unclassified;unclassified;unclassified;unclassified	1580	1364	80	797	1441	255	8	131	20	747	2	1	6	37
14	Bacteria;Bacteroidetes;Bacteroidia;Bacteroidales;[Paraprevotellaceae];[Prevotella];unclassified	1406	0	68	1	0	1	0	3865	0	313	7	0	1	1
15	Bacteria;Bacteroidetes;Bacteroidia;Bacteroidales;Prevotellaceae;Prevotella;copri	1154	0	4	5	4	55	1	113	187	2	7	0	3	0
16	Bacteria;Bacteroidetes;Bacteroidia;Bacteroidales;Prevotellaceae;Prevotella;stercorea	1126	130	14	63	0	0	0	1852	166	28	13	2	1	1
17	Bacteria;Firmicutes;Clostridia;Clostridiales;unclassified;unclassified;unclassified	1116	623	156	1249	335	519	6	23	198	883	809	6	2	14
18	Bacteria;Firmicutes;unclassified;unclassified;unclassified;unclassified;unclassified	978	3424	320	9285	2064	1990	6	1050	44	1415	7708	26	112	37
19	Bacteria;Bacteroidetes;Bacteroidia;Bacteroidales;Bacteroidaceae;Bacteroides;unclassified	851	179	10	47	62	83	14	147	8	137	61	43	221	31
20	Bacteria;Bacteroidetes;Bacteroidia;Bacteroidales;Prevotellaceae;Prevotella;copri	775	5	675	60	70	671	0	18	52	43	13	0	2	20
21	Bacteria;Actinobacteria;Actinobacteria;Bifidobacteriales;Bifidobacteriaceae;Bifidobacterium;bifidum	758	282	16	237	20	73	45	2586	9	7	6048	18097	5531	4360
22	Bacteria;Bacteroidetes;Bacteroidia;Bacteroidales;Rikenellaceae;Alistipes;putredinis	618	72	39	182	25	99	27	93	5	110	212	390	68	7
23	Bacteria;Firmicutes;Clostridia;Clostridiales;Ruminococcaceae;unclassified;unclassified	615	30	0	8	48	77	3	0	0	96	124	1	0	2
24	Bacteria;Bacteroidetes;Bacteroidia;Bacteroidales;Porphyromonadaceae;Parabacteroides;distasonis	539	27	23	44	22	79	20	10	812	8	26	488	6	16
25	Bacteria;Firmicutes;Clostridia;Clostridiales;unclassified;unclassified;unclassified	531	852	118	862	328	94	13	146	21	427	517	15	105	17
26	Bacteria;Bacteroidetes;Bacteroidia;Bacteroidales;Prevotellaceae;Prevotella;unclassified	488	1	100	4	2	0	2	1	546	71	0	0	1	9
27	Bacteria;unclassified;unclassified;unclassified;unclassified;unclassified;unclassified	424	1262	0	24	4	15	6	207	0	305	7	92	10	12
28	Bacteria;Firmicutes;Clostridia;Clostridiales;Ruminococcaceae;Oscillospira;unclassified	412	470	139	1670	643	264	9	29	100	426	211	34	10	17
29	Bacteria;Bacteroidetes;Bacteroidia;Bacteroidales;Bacteroidaceae;Bacteroides;unclassified	381	531	51	193	174	134	35	659	39	745	141	268	1099	81
30	Bacteria;unclassified;unclassified;unclassified;unclassified;unclassified;unclassified	371	13344	58	159	363	57	11	6	11	782	1056	8	34	8
31	Bacteria;Bacteroidetes;Bacteroidia;Bacteroidales;unclassified;unclassified;unclassified	363	1	0	269	2	1	4	0	238	50	1	102	4	0
32	Bacteria;unclassified;unclassified;unclassified;unclassified;unclassified;unclassified	342	2691	17	1833	209	1764	5	1	26	657	2441	1	47	14
33	Bacteria;unclassified;unclassified;unclassified;unclassified;unclassified;unclassified	318	975	57	1637	943	704	9	136	26	621	2453	11	2	28
34	Bacteria;Bacteroidetes;Bacteroidia;Bacteroidales;[Barnesiellaceae];unclassified;unclassified	299	30	7	3	37	412	0	13	0	1	103	133	8	0
35	Bacteria;Bacteroidetes;Bacteroidia;Bacteroidales;Bacteroidaceae;Bacteroides;ovatus	299	28	15	31	23	232	2	7	0	1	0	16	13	1
36	Bacteria;Bacteroidetes;Bacteroidia;Bacteroidales;Bacteroidaceae;Bacteroides;fragilis	273	24	642	354	508	1259	292	1671	345	26	1170	1454	253	907
37	Bacteria;Bacteroidetes;Bacteroidia;Bacteroidales;[Paraprevotellaceae];[Prevotella];unclassified	273	3	0	0	2	1	0	8	129	0	2	0	8	2
38	Bacteria;Bacteroidetes;Bacteroidia;Bacteroidales;Prevotellaceae;Prevotella;unclassified	258	0	0	2	1	2	0	58	153	26	0	0	0	0
39	Bacteria;Firmicutes;Clostridia;Clostridiales;Ruminococcaceae;unclassified;unclassified	258	102	35	19	167	334	0	417	489	221	916	5	874	17
40	Bacteria;Firmicutes;Clostridia;Clostridiales;Lachnospiraceae;unclassified;unclassified	257	69	151	110	234	474	8	44	1290	91	1263	24	212	30
41	Bacteria;Bacteroidetes;Bacteroidia;Bacteroidales;Rikenellaceae;unclassified;unclassified	242	8	17	90	33	101	3	6	2	4	60	2	1	1
42	Bacteria;Actinobacteria;Actinobacteria;Bifidobacteriales;Bifidobacteriaceae;Bifidobacterium;breve	236	20	22	27	3	9	5	27	0	1	4	2218	5003	146
43	Bacteria;Bacteroidetes;Bacteroidia;Bacteroidales;[Paraprevotellaceae];Paraprevotella;unclassified	229	23	7	45	11	450	0	32	2	0	0	2	1	0
44	Bacteria;Firmicutes;Clostridia;Clostridiales;unclassified;unclassified;unclassified	219	28	0	1	16	581	0	32	0	508	5	3	1	1
45	Bacteria;Bacteroidetes;Bacteroidia;Bacteroidales;Rikenellaceae;unclassified;unclassified	215	1	1	4	14	19	1	0	0	3	10	519	3	0
46	Bacteria;Bacteroidetes;Bacteroidia;Bacteroidales;[Paraprevotellaceae];[Prevotella];unclassified	213	0	0	361	1	0	0	0	103	0	0	2	0	1
47	Bacteria;Bacteroidetes;Bacteroidia;Bacteroidales;Prevotellaceae;Prevotella;copri	204	0	0	1	0	0	0	1	61	0	0	0	0	0
48	Bacteria;unclassified;unclassified;unclassified;unclassified;unclassified;unclassified	202	400	38	478	576	364	1	369	8	226	1703	79	40	12
49	Bacteria;Proteobacteria;Gammaproteobacteria;Aeromonadales;Succinivibrionaceae;Succinivibrio;unclas	199	0	0	0	0	633	0	11302	1	0	0	5	1	0
50	Bacteria;Firmicutes;Clostridia;Clostridiales;Lachnospiraceae;unclassified;unclassified	175	104	43	546	45	44	135	21	77	59	230	81	217	159
51	Bacteria;Bacteroidetes;Bacteroidia;Bacteroidales;[Paraprevotellaceae];[Prevotella];unclassified	173	0	5	0	0	0	0	106	0	0	0	0	0	0
52	Bacteria;Bacteroidetes;Bacteroidia;Bacteroidales;Bacteroidaceae;Bacteroides;unclassified	162	31	0	0	3	8	0	0	0	0	263	0	0	0
53	Bacteria;Verrucomicrobia;Verrucomicrobiae;Verrucomicrobiales;Verrucomicrobiaceae;Akkermansia;muci	161	93	254	54	5	279	20	3	8	57	219	2672	8	10
54	Bacteria;Actinobacteria;Coriobacteriia;Coriobacteriales;Coriobacteriaceae;unclassified;unclassified	140	12	26	145	152	74	30	402	80	72	277	1670	39	4
55	Bacteria;Bacteroidetes;Bacteroidia;Bacteroidales;Prevotellaceae;Prevotella;stercorea	139	0	2	0	0	0	0	18	7	0	0	0	0	0
56	Bacteria;Bacteroidetes;Bacteroidia;Bacteroidales;Bacteroidaceae;unclassified;unclassified	138	0	0	0	0	0	1	0	0	0	0	174	0	0
57	Bacteria;Bacteroidetes;Bacteroidia;Bacteroidales;unclassified;unclassified;unclassified	136	0	0	0	0	1	2	0	3	0	15	0	0	0
58	Bacteria;Firmicutes;Clostridia;Clostridiales;Christensenellaceae;unclassified;unclassified	132	723	0	880	8	3	1	6	8	204	4	4	8	25
59	Bacteria;unclassified;unclassified;unclassified;unclassified;unclassified;unclassified	132	425	5	658	40	12	2	1	40	303	6350	0	0	1
60	Bacteria;unclassified;unclassified;unclassified;unclassified;unclassified;unclassified	131	5	0	0	0	0	0	0	0	0	0	4	0	0
61	Bacteria;Firmicutes;Clostridia;Clostridiales;unclassified;unclassified;unclassified	127	1134	1	638	72	187	1	2	1	127	31	3	3	2
62	Bacteria;Actinobacteria;Coriobacteriia;Coriobacteriales;Coriobacteriaceae;unclassified;unclassified	122	511	29	52	13	193	10	206	11	95	1520	58	12	350
63	Bacteria;Firmicutes;Clostridia;Clostridiales;Ruminococcaceae;Oscillospira;unclassified	122	38	26	298	20	19	107	32	22	26	37	95	43	85
64	Bacteria;unclassified;unclassified;unclassified;unclassified;unclassified;unclassified	119	33	55	18	48	48	3	4	12	22	8	2	1	1
65	Bacteria;Bacteroidetes;unclassified;unclassified;unclassified;unclassified;unclassified	114	1	0	0	0	0	0	0	0	0	0	0	0	0
66	Bacteria;Bacteroidetes;Bacteroidia;Bacteroidales;unclassified;unclassified;unclassified	110	0	0	4	0	0	0	0	0	0	0	1	5	2
67	Bacteria;unclassified;unclassified;unclassified;unclassified;unclassified;unclassified	110	0	0	0	52	0	0	1	1	198	25	0	32	0
68	Bacteria;Bacteroidetes;Bacteroidia;Bacteroidales;S24-7;unclassified;unclassified	108	105	1648	1324	410	396	62	239	422	236	3127	2772	469	1155
69	Bacteria;Actinobacteria;Actinobacteria;Actinomycetales;Actinomycetaceae;Actinomyces;unclassified	104	6	7	132	19	9	1	30	28	38	121	5	15	387
70	Bacteria;Bacteroidetes;Bacteroidia;Bacteroidales;[Odoribacteraceae];Odoribacter;unclassified	98	11	1	6	31	21	0	3	4	1	23	266	1	0
71	Bacteria;unclassified;unclassified;unclassified;unclassified;unclassified;unclassified	95	457	8	124	105	58	1	0	1	11	10	2	1	7
72	Bacteria;Bacteroidetes;Bacteroidia;Bacteroidales;[Paraprevotellaceae];unclassified;unclassified	92	1	52	6	0	4	0	115	5	13	1	3	0	0
73	Bacteria;Bacteroidetes;Bacteroidia;Bacteroidales;[Paraprevotellaceae];[Prevotella];unclassified	90	0	0	0	0	0	0	1	0	0	0	0	0	0
74	Bacteria;Bacteroidetes;Bacteroidia;Bacteroidales;unclassified;unclassified;unclassified	89	0	0	0	0	0	0	0	0	0	0	0	0	1
75	Bacteria;Firmicutes;Clostridia;Clostridiales;unclassified;unclassified;unclassified	88	1	0	63	3	7	0	4	0	5	5	3	0	0
76	Bacteria;Bacteroidetes;Bacteroidia;Bacteroidales;Porphyromonadaceae;Parabacteroides;unclassified	88	25	0	22	1	2	0	3	2	0	2	1	1	0
77	Bacteria;Bacteroidetes;Bacteroidia;Bacteroidales;Bacteroidaceae;Bacteroides;coprophilus	82	0	0	0	0	0	0	0	0	2	0	0	241	0
78	Bacteria;Firmicutes;Clostridia;Clostridiales;unclassified;unclassified;unclassified	82	380	4	50	91	44	4	1	13	58	1	1	3	7
79	Bacteria;Bacteroidetes;Bacteroidia;Bacteroidales;unclassified;unclassified;unclassified	81	0	0	1	0	0	0	0	0	0	0	0	1	1
80	Bacteria;Firmicutes;Clostridia;Clostridiales;Christensenellaceae;unclassified;unclassified	78	273	6	2669	75	4	1	2	26	13	10	1	1	3
81	Bacteria;Firmicutes;Erysipelotrichi;Erysipelotrichales;Erysipelotrichaceae;Bulleidia;p-1630-c5	77	5	0	2	0	0	0	1	16	0	1	5	0	0
82	Bacteria;Firmicutes;Clostridia;Clostridiales;unclassified;unclassified;unclassified	74	50	5	104	20	21	0	1	0	12	3	2	1	0
83	Bacteria;Actinobacteria;Actinobacteria;Bifidobacteriales;Bifidobacteriaceae;Bifidobacterium;unclassified	74	6	9	315	4	5	76	2	2	4	5	33	6	8
84	Bacteria;Firmicutes;Clostridia;Clostridiales;Ruminococcaceae;Oscillospira;unclassified	71	3	6	5	4	2	1	0	1	8	3	0	0	1
85	Bacteria;Firmicutes;Clostridia;Clostridiales;unclassified;unclassified;unclassified	68	35	52	5	46	24	0	1	0	53	75	1	0	4
86	Bacteria;Firmicutes;Clostridia;Clostridiales;Christensenellaceae;unclassified;unclassified	67	248	0	101	99	10	4	1	8	40	2	0	0	1
87	Bacteria;Actinobacteria;Coriobacteriia;Coriobacteriales;Coriobacteriaceae;unclassified;unclassified	67	3	26	9	8	2	1	0	43	75	12	195	154	1
88	Bacteria;Bacteroidetes;Bacteroidia;Bacteroidales;Bacteroidaceae;Bacteroides;plebeius	64	81	0	1943	40	13	0	0	0	1	0	0	98	2
89	Bacteria;unclassified;unclassified;unclassified;unclassified;unclassified;unclassified	64	327	11	3359	310	250	2	2	4	40	199	0	1	5
90	Bacteria;unclassified;unclassified;unclassified;unclassified;unclassified;unclassified	63	2	4	16	11	1	1	1	1	9	9	3	0	0

91	Bacteria;Firmicutes;Clostridia;Clostridiales;Lachnospiraceae;Butyrivibrio;unclassified	62	0	1	1	1	0	0	1	0	0	1	7	0	1
92	Bacteria;Bacteroidetes;Bacteroidia;Bacteroidales;S24-7;unclassified;unclassified	61	34	621	211	15	74	4	83	16	49	2554	568	68	44
93	Bacteria;unclassified;unclassified;unclassified;unclassified;unclassified;unclassified	60	279	26	7	345	249	5	149	45	142	161	25	2	16
94	Bacteria;Bacteroidetes;Bacteroidia;Bacteroidales;[Paraprevotellaceae];Paraprevotella;unclassified	58	0	0	15	0	0	0	0	0	0	0	0	18	0
95	Bacteria;Actinobacteria;Coriobacteriia;Coriobacteriales;Coriobacteriaceae;unclassified;unclassified	56	10	0	182	8	1	0	1	0	0	2	13	88	0
96	Bacteria;Bacteroidetes;Bacteroidia;Bacteroidales;unclassified;unclassified;unclassified	55	0	0	2	0	2	0	0	6	6	0	0	1	4
97	Bacteria;Bacteroidetes;Bacteroidia;Bacteroidales;S24-7;unclassified;unclassified	51	106	8	780	13	123	88	116	23	12	21	65	752	13
98	Bacteria;Firmicutes;Clostridia;Clostridiales;unclassified;unclassified;unclassified	49	128	6	76	85	63	2	8	10	57	37	2	0	1
99	Bacteria;Bacteroidetes;Bacteroidia;Bacteroidales;Prevotellaceae;Prevotella;copri	49	0	2	0	1	9	0	5	22	2	7	0	0	0
100	Bacteria;unclassified;unclassified;unclassified;unclassified;unclassified;unclassified	47	119	0	69	355	6	0	2	1	14	1	0	2	7
101	Bacteria;unclassified;unclassified;unclassified;unclassified;unclassified;unclassified	45	63	9	19	7	2	0	0	1	12	0	0	0	0
102	Bacteria;Bacteroidetes;Bacteroidia;Bacteroidales;Bacteroidaceae;Bacteroides;caccae	45	0	0	0	0	0	0	0	0	0	0	0	0	0
103	Bacteria;unclassified;unclassified;unclassified;unclassified;unclassified;unclassified	45	2	0	13	1	53	1	28	0	1	34	1	27	0
104	Bacteria;Bacteroidetes;Bacteroidia;Bacteroidales;unclassified;unclassified;unclassified	43	3	1	35	0	23	12	0	3	0	4	6	0	3
105	Bacteria;unclassified;unclassified;unclassified;unclassified;unclassified;unclassified	41	9	11	35	4	13	1	36	4	0	416	5	2	1
106	Bacteria;Firmicutes;Clostridia;Clostridiales;unclassified;unclassified;unclassified	41	55	14	138	238	41	0	26	6	23	112	0	4	3
107	Bacteria;Firmicutes;Clostridia;Clostridiales;unclassified;unclassified;unclassified	40	5	0	17	5	7	0	0	0	2	0	151	1	0
108	Bacteria;Bacteroidetes;Bacteroidia;Bacteroidales;Rikenellaceae;Alistipes;indistinctus	37	3	0	8	3	10	0	0	0	0	1	0	0	0
109	Bacteria;Bacteroidetes;Bacteroidia;Bacteroidales;Prevotellaceae;Prevotella;copri	36	1	3	9	0	1	0	2	0	0	3	0	1	0
110	Bacteria;unclassified;unclassified;unclassified;unclassified;unclassified;unclassified	35	0	0	0	0	0	0	0	0	0	10	1	0	0
111	Bacteria;Firmicutes;Clostridia;Clostridiales;Ruminococcaceae;Oscillospira;unclassified	34	14	148	44	5	13	6	17	2	4	7	90	18	7
112	Bacteria;unclassified;unclassified;unclassified;unclassified;unclassified;unclassified	33	83	0	529	0	0	0	0	3	34	0	0	1	0
113	Bacteria;Bacteroidetes;Bacteroidia;Bacteroidales;Prevotellaceae;Prevotella;copri	33	0	0	0	2	11	0	2	4	0	1	1	0	0
114	Bacteria;Bacteroidetes;unclassified;unclassified;unclassified;unclassified;unclassified	29	0	0	67	0	0	0	0	0	6	3	0	1	0
115	Bacteria;Bacteroidetes;Bacteroidia;Bacteroidales;S24-7;unclassified;unclassified	28	0	0	0	0	0	0	0	21	0	20	10	2	0
116	Bacteria;Actinobacteria;Actinobacteria;Actinomycetales;Micrococcaceae;Rothia;mucilaginosa	28	3	6	1	4	9	2	2	16	26	129	13	4	2904
117	Bacteria;Actinobacteria;unclassified;unclassified;unclassified;unclassified;unclassified	27	0	1	46	0	25	1	52	0	1	64	235	47	1
118	Bacteria;Bacteroidetes;Bacteroidia;Bacteroidales;[Paraprevotellaceae];CF231;unclassified	26	0	0	0	0	0	0	33	5	0	0	0	0	0
119	Bacteria;Cyanobacteria;4C0d-2;YS2;unclassified;unclassified;unclassified	26	3	4	277	591	243	5	2	4	57	8	4	3	19
120	Bacteria;Bacteroidetes;Bacteroidia;Bacteroidales;S24-7;unclassified;unclassified	25	26	266	184	53	111	7	12	62	7	30	991	35	126
121	Bacteria;Bacteroidetes;Bacteroidia;Bacteroidales;Bacteroidaceae;Bacteroides;fragilis	25	17	6	2	8	16	27	83	5	0	8	7	1	1
122	Bacteria;unclassified;unclassified;unclassified;unclassified;unclassified;unclassified	25	76	3	51	28	98	0	0	1	8	5	0	1	1
123	Bacteria;unclassified;unclassified;unclassified;unclassified;unclassified;unclassified	24	0	0	1	0	0	0	4	0	0	0	0	0	0
124	Bacteria;Bacteroidetes;Bacteroidia;Bacteroidales;Bacteroidaceae;Bacteroides;unclassified	24	0	0	1	4	0	0	1	0	0	0	0	3	0
125	Bacteria;Actinobacteria;Coriobacteriia;Coriobacteriales;Coriobacteriaceae;unclassified;unclassified	24	57	15	1	5	6	1	0	205	70	165	65	1	3
126	Bacteria;Actinobacteria;Actinobacteria;Bifidobacteriales;Bifidobacteriaceae;unclassified;unclassified	23	2	0	50	2	79	4	1	1	0	19	3	165	1
127	Bacteria;unclassified;unclassified;unclassified;unclassified;unclassified;unclassified	23	6	6	45	7	2	0	8	0	8	22	0	1	0
128	Bacteria;Actinobacteria;Coriobacteriia;Coriobacteriales;Coriobacteriaceae;Slackia;unclassified	23	452	53	932	125	157	2	246	484	155	506	296	1	9
129	Bacteria;unclassified;unclassified;unclassified;unclassified;unclassified;unclassified	22	0	0	0	0	28	0	8	0	0	3	1	2	0
130	Bacteria;Firmicutes;Clostridia;Clostridiales;unclassified;unclassified;unclassified	22	39	29	56	4	5	6	0	0	0	0	0	0	0
131	Bacteria;Bacteroidetes;Bacteroidia;Bacteroidales;unclassified;unclassified;unclassified	22	0	31	0	0	0	1	0	106	0	0	0	0	0
132	Bacteria;Bacteroidetes;Bacteroidia;Bacteroidales;[Paraprevotellaceae];[Prevotella];unclassified	22	0	0	0	0	0	1	0	0	0	0	0	0	0
133	Bacteria;Bacteroidetes;Bacteroidia;Bacteroidales;S24-7;unclassified;unclassified	22	34	6	80	15	22	17	406	12	281	23	21	175	17
134	Bacteria;unclassified;unclassified;unclassified;unclassified;unclassified;unclassified	22	116	0	6	2	7	1	0	1	8	0	1	1	0
135	Bacteria;Bacteroidetes;Bacteroidia;Bacteroidales;Prevotellaceae;Prevotella;copri	20	0	2	1	0	5	0	4	3	0	0	0	0	0
136	Bacteria;Actinobacteria;unclassified;unclassified;unclassified;unclassified;unclassified	19	1	2	35	2	3	0	18	0	1	59	3	5	0
137	Bacteria;Bacteroidetes;Bacteroidia;Bacteroidales;Prevotellaceae;Prevotella;copri	17	0	0	0	1	0	0	36	1	0	0	0	0	0
138	Bacteria;Bacteroidetes;Bacteroidia;Bacteroidales;S24-7;unclassified;unclassified	17	13	5	175	8	68	48	178	8	86	21	23	97	15
139	Bacteria;Bacteroidetes;Bacteroidia;Bacteroidales;unclassified;unclassified;unclassified	16	1	0	0	0	3	0	2	0	0	0	0	0	0
140	Bacteria;unclassified;unclassified;unclassified;unclassified;unclassified;unclassified	16	0	0	547	0	0	0	2	0	109	6	0	4	0
141	Bacteria;Fusobacteria;Fusobacteriia;Fusobacteriales;Fusobacteriaceae;Fusobacterium;unclassified	14	1	0	2	0	9	0	409	0	17	1	24	1	1
142	Bacteria;Firmicutes;Clostridia;Clostridiales;unclassified;unclassified;unclassified	14	2	0	37	9	35	1	0	1	12	49	0	0	1
143	Bacteria;Firmicutes;Clostridia;Clostridiales;unclassified;unclassified;unclassified	14	2	56	79	5	0	1	5	235	4	18	3	0	3
144	Bacteria;Bacteroidetes;Bacteroidia;Bacteroidales;Rikenellaceae;unclassified;unclassified	14	70	5	23	10	15	2	5	3	0	6	6	610	1
145	Bacteria;Bacteroidetes;Bacteroidia;Bacteroidales;S24-7;unclassified;unclassified	14	29	2	147	7	62	14	7	1	6	19	15	356	15
146	Bacteria;unclassified;unclassified;unclassified;unclassified;unclassified;unclassified	14	1	4	50	2	1	0	8	3	0	124	10	10	0
147	Bacteria;Firmicutes;Clostridia;Clostridiales;Ruminococcaceae;unclassified;unclassified	13	1	0	1	1	0	1	0	0	0	355	0	2	0
148	Bacteria;Firmicutes;Clostridia;Clostridiales;unclassified;unclassified;unclassified	12	0	0	11567	1	0	0	0	0	12	3	2	0	0
149	Bacteria;unclassified;unclassified;unclassified;unclassified;unclassified;unclassified	12	196	24	0	83	23	0	1	250	106	584	401	2	0
150	Bacteria;Firmicutes;Clostridia;Clostridiales;Ruminococcaceae;Oscillospira;unclassified	12	1	6	7	15	6	3	0	1	0	25	8	7	1
151	Bacteria;Bacteroidetes;Bacteroidia;Bacteroidales;S24-7;unclassified;unclassified	11	30	5	68	8	18	10	12	2	15	19	15	24	12
152	Bacteria;unclassified;unclassified;unclassified;unclassified;unclassified;unclassified	11	57	10	25	16	17	0	0	0	8	4	0	0	0
153	Bacteria;Firmicutes;Clostridia;Clostridiales;Ruminococcaceae;unclassified;unclassified	11	0	0	1581	195	542	0	0	0	3	2	0	0	5
154	Bacteria;Bacteroidetes;unclassified;unclassified;unclassified;unclassified;unclassified	11	131	0	0	0	0	0	0	0	0	0	0	2	0
155	Bacteria;Actinobacteria;unclassified;unclassified;unclassified;unclassified;unclassified	10	0	0	0	0	0	0	20	0	0	1	0	0	0
156	Bacteria;Firmicutes;Clostridia;Clostridiales;Ruminococcaceae;Oscillospira;unclassified	10	17	29	35	59	15	1	2	8	41	3	4	11	2
157	Bacteria;unclassified;unclassified;unclassified;unclassified;unclassified;unclassified	10	4	4	8	5	22	0	0	0	3	10	0	0	1
158	Bacteria;unclassified;unclassified;unclassified;unclassified;unclassified;unclassified	9	0	0	5	0	0	0	0	1	3	167	0	0	0
159	Bacteria;Bacteroidetes;Bacteroidia;Bacteroidales;unclassified;unclassified;unclassified	9	0	30	0	0	0	0	268	0	0	0	1	0	0
160	Bacteria;Firmicutes;Clostridia;Clostridiales;Ruminococcaceae;Faecalibacterium;prausnitzii	9	6	2	2	2	17	0	17	3	10	138	0	2	0
161	Bacteria;unclassified;unclassified;unclassified;unclassified;unclassified;unclassified	8	6	0	5	2	0	4	0	1866	9	309	0	0	18
162	Bacteria;Firmicutes;Clostridia;Clostridiales;Eubacteriaceae;Pseudoramibacter_Eubacterium;unclassified	8	1	0	16	0	2	1	1	0	1	0	4914	1	1
163	Bacteria;Firmicutes;Clostridia;Clostridiales;Christensenellaceae;unclassified;unclassified	8	2	0	65	0	0	0	0	0	4	0	0	0	0
164	Bacteria;Bacteroidetes;Bacteroidia;Bacteroidales;S24-7;unclassified;unclassified	8	13	8	20	5	1	4	253	3	192	28	9	147	16
165	Bacteria;Firmicutes;Clostridia;Clostridiales;Ruminococcaceae;unclassified;unclassified	8	0	0	0	0	1	0	0	23	0	2	8	0	1
166	Bacteria;Bacteroidetes;Bacteroidia;Bacteroidales;S24-7;unclassified;unclassified	8	25	5	155	12	31	28	126	4	94	9	19	93	5
167	Bacteria;Firmicutes;Clostridia;Clostridiales;Ruminococcaceae;unclassified;unclassified	8	0	0	55	0	3	0	0	0	0	4	0	1	0
168	Bacteria;Bacteroidetes;Bacteroidia;Bacteroidales;[Odoribacteraceae];Butyricimonas;unclassified	8	15	1	15	6	104	0	0	0	1	35	0	0	0
169	Bacteria;Firmicutes;Clostridia;Clostridiales;Lachnospiraceae;unclassified;unclassified	8	0	0	15	0	4	0	11	2	2	29	20	8	1
170	Bacteria;unclassified;unclassified;unclassified;unclassified;unclassified;unclassified	8	0	0	12	2	16	0	0	0	2	23	0	0	0
171	Bacteria;unclassified;unclassified;unclassified;unclassified;unclassified;unclassified	7	42	0	31	92	0	0	0	2	0	0	0	0	3
172	Bacteria;unclassified;unclassified;unclassified;unclassified;unclassified;unclassified	7	5	0	22	11	1	0	2	0	4	1	0	0	0
173	Bacteria;Proteobacteria;Alphaproteobacteria;Rickettsiales;mitochondria;unclassified;unclassified	7	24	1	57	5	16	5	22	9	9	94	6	20	19
174	Bacteria;Bacteroidetes;Bacteroidia;Bacteroidales;unclassified;unclassified;unclassified	7	0	0	0	0	0	0	25	0	0	0	0	0	0
175	Bacteria;Bacteroidetes;Bacteroidia;Bacteroidales;Bacteroidaceae;Bacteroides;eggerthii	6	0	1	0	0	0	0	0	0	0	30	0	0	0
176	Bacteria;Actinobacteria;Actinobacteria;Bifidobacteriales;Bifidobacteriaceae;unclassified;unclassified	6	1	0	3	1	19	1	2	1	0	8	28	44	1
177	Bacteria;Firmicutes;Clostridia;Clostridiales;Ruminococcaceae;unclassified;unclassified	6	1	0	61	0	1	0	0	0	1	0	0	0	0
178	Bacteria;Verrucomicrobia;Verrucomicrobiae;Verrucomicrobiales;Verrucomicrobiaceae;unclassified;uncla	6	0	6	0	2	11	0	0	0	0	21	20	1	0
179	Bacteria;unclassified;unclassified;unclassified;unclassified;unclassified;unclassified	6	51	0	0	0	0	1	0	0	0	0	0	0	0
180	Bacteria;Firmicutes;unclassified;unclassified;unclassified;unclassified;unclassified	6	1	38	1	1	2	0	0	0	0	0	0	0	0

181	Bacteria;Firmicutes;Clostridia;unclassified;unclassified;unclassified;unclassified	6	66	1	24	8	2	0	1	0	9	11	0	0	0
182	Bacteria;Firmicutes;Clostridia;Clostridiales;Lachnospiraceae;unclassified;unclassified	6	10	58	91	2	15	8	6	3	6	6	28	14	9
183	Bacteria;Bacteroidetes;Bacteroidia;Bacteroidales;unclassified;unclassified;unclassified	6	0	0	0	18	41	0	0	0	0	3	0	0	0
184	Bacteria;unclassified;unclassified;unclassified;unclassified;unclassified;unclassified	6	17	0	0	22	0	0	0	0	0	0	1	0	0
185	Bacteria;unclassified;unclassified;unclassified;unclassified;unclassified;unclassified	5	3	0	0	0	1	1	28	5	0	10	1	14	2
186	Bacteria;Bacteroidetes;Bacteroidia;Bacteroidales;S24-7;unclassified;unclassified	5	17	1	14	3	4	6	7	4	26	10	8	179	7
187	Bacteria;Actinobacteria;Coriobacteriia;Coriobacteriales;Coriobacteriaceae;Eggerthella;lenta	5	0	0	10	1	0	0	1	0	0	12	0	38	0
188	Bacteria;Firmicutes;Clostridia;Clostridiales;Lachnospiraceae;unclassified;unclassified	5	0	0	3	0	0	0	8	0	4	21	2	5	0
189	Bacteria;Bacteroidetes;Bacteroidia;Bacteroidales;S24-7;unclassified;unclassified	5	4	2	309	1	65	29	2	17	4	6	20	11	8
190	Bacteria;Actinobacteria;unclassified;unclassified;unclassified;unclassified;unclassified	5	1	0	8	2	1	0	1	3	0	32	3	0	45
191	Bacteria;unclassified;unclassified;unclassified;unclassified;unclassified;unclassified	5	37	0	11	2	7	0	0	1	15	0	0	0	0
192	Bacteria;unclassified;unclassified;unclassified;unclassified;unclassified;unclassified	5	3	1	41	15	7	2	1	0	2	2	3	3	0
193	Bacteria;Bacteroidetes;Bacteroidia;Bacteroidales;S24-7;unclassified;unclassified	5	6	4	88	1	23	19	137	5	121	8	13	46	7
194	Bacteria;unclassified;unclassified;unclassified;unclassified;unclassified;unclassified	5	163	0	79	0	0	3	0	0	16	0	0	0	0
195	Bacteria;Firmicutes;Clostridia;Clostridiales;Ruminococcaceae;Clostridium;unclassified	5	0	4	0	5	5	0	0	1	1	22	0	1	0
196	Bacteria;unclassified;unclassified;unclassified;unclassified;unclassified;unclassified	5	0	0	21	0	11	0	0	0	0	9	0	1	0
197	Bacteria;Bacteroidetes;Bacteroidia;Bacteroidales;S24-7;unclassified;unclassified	5	19	5	18	1	7	1	4	1	10	3	13	104	4
198	Bacteria;unclassified;unclassified;unclassified;unclassified;unclassified;unclassified	5	1	1	62	2	4	0	10	0	2	15	5	0	1
199	Bacteria;Firmicutes;Clostridia;Clostridiales;Lachnospiraceae;unclassified;unclassified	5	10	0	20	2	14	9	1	9	1	18	3	5	122
200	Bacteria;Bacteroidetes;Bacteroidia;Bacteroidales;S24-7;unclassified;unclassified	5	5	2	6	2	1	2	33	3	31	7	6	14	8
201	Bacteria;Proteobacteria;Epsilonproteobacteria;Campylobacterales;Campylobacteraceae;Campylobacter;	5	0	0	2	128	692	0	0	2	0	5	0	0	1
202	Bacteria;unclassified;unclassified;unclassified;unclassified;unclassified;unclassified	5	51	4	5	0	0	0	1	4	5	4	0	1	0
203	Bacteria;Bacteroidetes;Bacteroidia;Bacteroidales;S24-7;unclassified;unclassified	5	17	2	27	6	22	9	2	3	1	4	20	277	2
204	Bacteria;Bacteroidetes;Bacteroidia;Bacteroidales;unclassified;unclassified;unclassified	5	0	0	0	0	0	0	0	0	0	0	0	30	0
205	Bacteria;unclassified;unclassified;unclassified;unclassified;unclassified;unclassified	4	5	6	3	8	0	1	9	23	0	18	5	0	129
206	Bacteria;unclassified;unclassified;unclassified;unclassified;unclassified;unclassified	4	0	1	21	1	1	0	1	3	0	9	5	0	1
207	Bacteria;Bacteroidetes;Bacteroidia;Bacteroidales;S24-7;unclassified;unclassified	4	3	0	109	0	63	33	4	14	1	5	21	6	5
208	Bacteria;unclassified;unclassified;unclassified;unclassified;unclassified;unclassified	4	56	0	0	0	0	0	0	0	0	1	0	2	0
209	Bacteria;Bacteroidetes;Bacteroidia;Bacteroidales;S24-7;unclassified;unclassified	4	10	0	103	0	22	13	5	5	2	2	6	51	0
210	Bacteria;Bacteroidetes;Bacteroidia;Bacteroidales;S24-7;unclassified;unclassified	4	4	45	14	13	12	2	10	12	7	16	68	13	15
211	Bacteria;Bacteroidetes;Bacteroidia;Bacteroidales;S24-7;unclassified;unclassified	4	17	1	35	3	9	13	16	3	8	7	10	65	1
212	Bacteria;unclassified;unclassified;unclassified;unclassified;unclassified;unclassified	4	48	5	31	5	11	6	41	11	1	135	9	45	11
213	Bacteria;Firmicutes;Clostridia;Clostridiales;unclassified;unclassified;unclassified	4	6	10	11	1	47	0	3	0	0	14	1	4	1
214	Bacteria;Actinobacteria;Actinobacteria;Bifidobacteriales;Bifidobacteriaceae;unclassified;unclassified	4	1	0	1	0	1	0	3	0	0	25	0	5	0
215	Bacteria;Bacteroidetes;Bacteroidia;Bacteroidales;S24-7;unclassified;unclassified	4	10	0	10	1	11	5	17	1	1	7	7	200	2
216	Bacteria;Firmicutes;Clostridia;Clostridiales;unclassified;unclassified;unclassified	3	1034	1	1	0	0	0	0	0	0	0	0	0	0
217	Bacteria;Firmicutes;Clostridia;Clostridiales;Lachnospiraceae;unclassified;unclassified	3	2	106	5	0	5	1	6	1	2	5	18	6	2
218	Bacteria;unclassified;unclassified;unclassified;unclassified;unclassified;unclassified	3	9	22	50	9	69	2	134	2	19	5	14	84	5
219	Bacteria;Firmicutes;Clostridia;Clostridiales;Lachnospiraceae;Clostridium;unclassified	3	0	0	4	0	0	0	0	1	0	456	0	9	3
220	Bacteria;Bacteroidetes;Bacteroidia;Bacteroidales;S24-7;unclassified;unclassified	3	18	1	82	0	12	14	4	2	1	6	10	249	3
221	Bacteria;Bacteroidetes;Bacteroidia;Bacteroidales;S24-7;unclassified;unclassified	3	4	2	8	1	0	1	252	0	109	27	2	174	3
222	Bacteria;unclassified;unclassified;unclassified;unclassified;unclassified;unclassified	3	1	2	7	1	5	0	2	3	0	30	0	0	6
223	Bacteria;Proteobacteria;Gammaproteobacteria;unclassified;unclassified;unclassified;unclassified	3	0	0	0	2	0	0	7	3	0	15	1	2	52
224	Bacteria;Bacteroidetes;Bacteroidia;Bacteroidales;S24-7;unclassified;unclassified	3	21	1	107	5	19	18	3	5	2	9	12	51	2
225	Bacteria;Firmicutes;Clostridia;unclassified;unclassified;unclassified;unclassified	3	0	0	0	0	0	1	119	0	0	1	0	0	0
226	Bacteria;unclassified;unclassified;unclassified;unclassified;unclassified;unclassified	3	39	0	26	0	16	0	0	0	0	63	0	0	0
227	Bacteria;Firmicutes;Clostridia;Clostridiales;Christensenellaceae;unclassified;unclassified	3	15	0	30	2	1	0	0	0	2	3	0	0	0
228	Bacteria;Firmicutes;Clostridia;Clostridiales;Ruminococcaceae;unclassified;unclassified	3	1	0	0	0	1	0	6	5	0	26	0	0	0
229	Bacteria;Cyanobacteria;4C0d-2;YS2;unclassified;unclassified;unclassified	3	0	0	0	0	0	0	0	0	24	0	0	0	1
230	Bacteria;Fusobacteria;Fusobacteriia;Fusobacteriales;Fusobacteriaceae;Cetobacterium;somerae	3	0	0	1	0	0	0	3101	0	0	0	0	0	0
231	Bacteria;Bacteroidetes;Bacteroidia;Bacteroidales;Bacteroidaceae;Bacteroides;unclassified	3	26	0	0	5	11	0	0	0	0	1	0	0	0
232	Bacteria;Firmicutes;Clostridia;Clostridiales;unclassified;unclassified;unclassified	2	4	1	0	51	10	0	2	1	3	2	0	0	1
233	Bacteria;unclassified;unclassified;unclassified;unclassified;unclassified;unclassified	2	0	0	0	0	0	0	0	0	0	849	0	0	0
234	Bacteria;unclassified;unclassified;unclassified;unclassified;unclassified;unclassified	2	64	0	0	0	0	1	0	0	201	0	0	0	0
235	Bacteria;Elusimicrobia;Elusimicrobia;Elusimicrobiales;Elusimicrobiaceae;unclassified;unclassified	2	0	0	1	5	68	0	0	2	0	0	0	0	0
236	Bacteria;Firmicutes;Clostridia;Clostridiales;Clostridiaceae;unclassified;unclassified	2	0	3	62	9	4	1	0	0	2	1	1	1	3
237	Bacteria;Bacteroidetes;Bacteroidia;Bacteroidales;S24-7;unclassified;unclassified	2	2	1	95	0	45	19	4	8	2	1	16	4	3
238	Bacteria;Firmicutes;Clostridia;Clostridiales;unclassified;unclassified;unclassified	2	9	2	90	9	0	0	0	1	1	2	0	0	0
239	Bacteria;unclassified;unclassified;unclassified;unclassified;unclassified;unclassified	2	1	31	32	1	6	3	0	0	14	94	93	0	1
240	Bacteria;unclassified;unclassified;unclassified;unclassified;unclassified;unclassified	2	34	0	81	0	0	0	0	0	0	0	0	0	0
241	Bacteria;unclassified;unclassified;unclassified;unclassified;unclassified;unclassified	2	0	0	0	0	7	0	29	0	0	0	0	0	0
242	Bacteria;unclassified;unclassified;unclassified;unclassified;unclassified;unclassified	2	0	0	0	0	0	0	148	0	0	0	0	0	0
243	Bacteria;unclassified;unclassified;unclassified;unclassified;unclassified;unclassified	2	0	0	67	5	1	0	0	9	0	4	18	1	12
244	Bacteria;Bacteroidetes;Bacteroidia;Bacteroidales;S24-7;unclassified;unclassified	2	7	1	5	1	4	0	2	1	0	4	0	64	0
245	Bacteria;Bacteroidetes;Bacteroidia;Bacteroidales;S24-7;unclassified;unclassified	2	19	0	3	1	1	1	0	1	0	3	2	280	0
246	Bacteria;Firmicutes;Clostridia;Clostridiales;unclassified;unclassified;unclassified	2	2	1	61	5	3	0	0	1	1	2	0	2	2
247	Bacteria;Firmicutes;Clostridia;Clostridiales;unclassified;unclassified;unclassified	2	0	0	20	2	8	0	1	0	0	9	0	0	0
248	Bacteria;unclassified;unclassified;unclassified;unclassified;unclassified;unclassified	2	0	0	0	0	1	0	36	1	4	14	4	1	0
249	Bacteria;Firmicutes;unclassified;unclassified;unclassified;unclassified;unclassified	2	0	1	4	1	15	0	3	1	1	28	0	0	0
250	Bacteria;Actinobacteria;Actinobacteria;Actinomycetales;Corynebacteriaceae;Corynebacterium;durum	2	1	1	32	6	1	0	1	1	1	3	0	0	1
251	Bacteria;unclassified;unclassified;unclassified;unclassified;unclassified;unclassified	2	0	0	1	0	19	0	0	0	0	0	3	21	0
252	Bacteria;Bacteroidetes;Bacteroidia;Bacteroidales;S24-7;unclassified;unclassified	2	1	0	45	0	23	9	0	0	0	2	4	2	0
253	Bacteria;Bacteroidetes;Bacteroidia;Bacteroidales;S24-7;unclassified;unclassified	2	12	0	4	0	7	3	2	0	11	3	0	111	2
254	Bacteria;Bacteroidetes;Bacteroidia;Bacteroidales;S24-7;unclassified;unclassified	2	1	0	77	3	13	10	0	4	2	3	8	3	1
255	Bacteria;Bacteroidetes;Bacteroidia;Bacteroidales;Prevotellaceae;Prevotella;unclassified	2	0	1	55	1	30	12	1	6	0	0	10	4	1
256	Bacteria;Actinobacteria;Actinobacteria;Actinomycetales;unclassified;unclassified;unclassified	2	0	0	3	0	0	0	1	0	0	1	49	1	18
257	Bacteria;Bacteroidetes;Bacteroidia;Bacteroidales;unclassified;unclassified;unclassified	2	2	7	7	19	6	0	3	6	11	17	24	12	10
258	Bacteria;Bacteroidetes;Bacteroidia;Bacteroidales;S24-7;unclassified;unclassified	2	0	1	25	0	7	1	0	0	0	1	2	1	0
259	Bacteria;Bacteroidetes;Bacteroidia;Bacteroidales;S24-7;unclassified;unclassified	2	5	0	24	0	3	4	2	3	3	1	2	13	1
260	Bacteria;Bacteroidetes;Bacteroidia;Bacteroidales;S24-7;unclassified;unclassified	1	0	24	4	1	2	2	0	1	0	155	14	0	2
261	Bacteria;Bacteroidetes;Bacteroidia;Bacteroidales;S24-7;unclassified;unclassified	1	2	29	10	1	2	0	1	3	0	0	14	0	5
262	Bacteria;unclassified;unclassified;unclassified;unclassified;unclassified;unclassified	1	0	1	20	0	0	0	0	0	0	1	1	0	0
263	Bacteria;Firmicutes;Clostridia;Clostridiales;Ruminococcaceae;Anaerotruncus;unclassified	1	4	6	3	0	0	0	2	0	0	204	3	14	0
264	Bacteria;Proteobacteria;Gammaproteobacteria;unclassified;unclassified;unclassified;unclassified	1	0	0	0	0	0	0	103	0	0	0	0	0	0
265	Bacteria;Actinobacteria;Coriobacteriia;Coriobacteriales;Coriobacteriaceae;Slackia;unclassified	1	0	0	118	0	0	0	0	0	0	0	0	0	0
266	Bacteria;Bacteroidetes;Bacteroidia;Bacteroidales;S24-7;unclassified;unclassified	1	0	0	38	0	22	12	2	1	0	5	14	3	2
267	Bacteria;unclassified;unclassified;unclassified;unclassified;unclassified;unclassified	1	53	0	0	0	0	0	0	0	0	0	0	0	0
268	Bacteria;Bacteroidetes;Bacteroidia;Bacteroidales;[Odoribacteraceae];Odoribacter;unclassified	1	7	0	7	1	4	2	2	0	0	1	1	55	1
269	Bacteria;unclassified;unclassified;unclassified;unclassified;unclassified;unclassified	1	0	13	8	5	2	1	0	6	1	11	26	1	47
270	Bacteria;unclassified;unclassified;unclassified;unclassified;unclassified;unclassified	1	0	31	2	0	1	3	18	0	6	1	3	18	1
271	Bacteria;Bacteroidetes;Bacteroidia;Bacteroidales;Porphyromonadaceae;unclassified;unclassified	1	1	0	0	0	1	1	3	0	0	24	0	0	0
272	Bacteria;unclassified;unclassified;unclassified;unclassified;unclassified;unclassified	1	0	0	16	1	0	0	0	11	0	0	14	0	55
273	Bacteria;Bacteroidetes;Bacteroidia;Bacteroidales;S24-7;unclassified;unclassified	1	0	1	21	0	13	10	0	1	0	1	4	1	0
274	Bacteria;unclassified;unclassified;unclassified;unclassified;unclassified;unclassified	1	0	0	42	0	0	0	0	0	0	0	0	0	0
275	Bacteria;Bacteroidetes;Bacteroidia;Bacteroidales;S24-7;unclassified;unclassified	1	1	1	1	1	0	0	92	1	20	4	2	54	0
276	Bacteria;Bacteroidetes;Bacteroidia;Bacteroidales;Bacteroidaceae;Bacteroides;unclassified	1	0	0	50	0	17	3	1	5	0	1	1	2	0
277	Bacteria;Bacteroidetes;Bacteroidia;Bacteroidales;unclassified;unclassified;unclassified	1	0	0	50	0	20	4	1	4	0	0	2	1	1
278	Bacteria;unclassified;unclassified;unclassified;unclassified;unclassified;unclassified	1	1	17	44	1	4	0	2	2	0	7	12	0	2
279	Bacteria;Fusobacteria;Fusobacteriia;Fusobacteriales;Leptotrichiaceae;Leptotrichia;unclassified	1	0	2	0	0	0	0	0	0	0	121	0	0	0
280	Bacteria;Bacteroidetes;Bacteroidia;Bacteroidales;Prevotellaceae;Prevotella;unclassified	1	0	0	0	0	0	0	1	0	0	15	4	0	24
281	Bacteria;unclassified;unclassified;unclassified;unclassified;unclassified;unclassified	1	22	0	0	0	1	0	0	8	9	0	0	0	0
282	Bacteria;unclassified;unclassified;unclassified;unclassified;unclassified;unclassified	1	5	4	0	0	6	0	0	0	5	107	0	0	0
283	Bacteria;unclassified;unclassified;unclassified;unclassified;unclassified;unclassified	1	1	0	6	7	1	0	0	10	1	9	30	0	9
284	Bacteria;Bacteroidetes;Bacteroidia;Bacteroidales;S24-7;unclassified;unclassified	1	0	0	37	1	18	6	0	0	0	0	0	1	1
285	Bacteria;Bacteroidetes;Bacteroidia;Bacteroidales;S24-7;unclassified;unclassified	1	4	0	13	0	10	3	0	0	0	2	1	37	1
286	Bacteria;Bacteroidetes;Bacteroidia;Bacteroidales;S24-7;unclassified;unclassified	1	0	0	41	0	7	9	1	1	0	0	4	0	0
287	Bacteria;Actinobacteria;Actinobacteria;unclassified;unclassified;unclassified;unclassified	1	0	0	3	0	0	0	4	0	0	7	29	0	0
288	Bacteria;Firmicutes;Clostridia;unclassified;unclassified;unclassified;unclassified	1	0	0	1	0	0	0	0	0	0	32	0	1	0
289	Bacteria;Bacteroidetes;unclassified;unclassified;unclassified;unclassified;unclassified	1	0	0	27	0	4	5	0	1	1	1	2	0	0
290	Bacteria;unclassified;unclassified;unclassified;unclassified;unclassified;unclassified	1	6	0	75	8	1	0	0	0	0	0	0	0	0
291	Bacteria;Bacteroidetes;Bacteroidia;Bacteroidales;unclassified;unclassified;unclassified	1	365	0	0	0	0	0	0	0	0	0	0	0	0
292	Bacteria;Bacteroidetes;Bacteroidia;Bacteroidales;Prevotellaceae;Prevotella;unclassified	1	1	0	0	1	0	0	25	2	1	6	0	0	0
293	Bacteria;Bacteroidetes;Bacteroidia;Bacteroidales;Prevotellaceae;Prevotella;unclassified	0	0	0	0	0	0	0	13	0	0	26	0	0	0
294	Bacteria;Bacteroidetes;Bacteroidia;Bacteroidales;S24-7;unclassified;unclassified	0	0	0	5	0	2	3	0	1	0	1	8	44	1
295	Bacteria;unclassified;unclassified;unclassified;unclassified;unclassified;unclassified	0	0	0	5	30	0	0	0	0	0	0	0	0	0
296	Bacteria;unclassified;unclassified;unclassified;unclassified;unclassified;unclassified	0	0	0	0	0	6	0	22	0	0	0	0	0	0
297	Bacteria;unclassified;unclassified;unclassified;unclassified;unclassified;unclassified	0	0	0	80	2	4	0	0	2	0	0	17	0	0
298	Bacteria;Bacteroidetes;Bacteroidia;Bacteroidales;S24-7;unclassified;unclassified	0	0	0	22	0	2	7	0	0	0	1	1	0	1
299	Bacteria;Actinobacteria;Actinobacteria;Actinomycetales;unclassified;unclassified;unclassified	0	0	0	0	0	28	0	0	0	0	1	0	0	0
300	Bacteria;Actinobacteria;Actinobacteria;Bifidobacteriales;Bifidobacteriaceae;Bifidobacterium;longum	0	1	0	2	0	0	0	1	1	0	1	1	0	79
301	Bacteria;Bacteroidetes;Bacteroidia;Bacteroidales;Porphyromonadaceae;Porphyromonas;unclassified	0	0	0	0	0	0	1	0	0	0	23	0	0	0
302	Bacteria;Actinobacteria;Actinobacteria;Bifidobacteriales;Bifidobacteriaceae;Bifidobacterium;longum	0	0	0	0	0	0	0	0	0	0	5	2	0	87
303	Bacteria;Bacteroidetes;Flavobacteriia;Flavobacteriales;[Weeksellaceae];unclassified;unclassified	0	0	0	0	0	0	0	0	0	80	0	0	0	0
304	Bacteria;unclassified;unclassified;unclassified;unclassified;unclassified;unclassified	0	0	0	31	0	0	0	0	0	0	0	0	0	0
305	Bacteria;Firmicutes;unclassified;unclassified;unclassified;unclassified;unclassified	0	1	0	32	0	0	0	1	1	0	2	0	0	0
306	Bacteria;unclassified;unclassified;unclassified;unclassified;unclassified;unclassified	0	0	0	1	0	0	0	0	0	0	21	0	0	0
307	Bacteria;Cyanobacteria;4C0d-2;YS2;unclassified;unclassified;unclassified	0	9	0	31	0	0	0	0	0	0	0	0	0	0
308	Bacteria;Actinobacteria;Actinobacteria;Bifidobacteriales;Bifidobacteriaceae;Bifidobacterium;longum	0	0	0	0	0	0	0	0	0	0	0	0	0	21
309	Bacteria;Firmicutes;Clostridia;unclassified;unclassified;unclassified;unclassified	0	0	0	21	0	0	0	0	0	0	0	0	0	0
