# Supplementary figures and images for: Fecal microbiota transplantation from protozoa-exposed donors downregulates immune response in a germ-free mouse model, its role in immune response and physiology of the intestine
Source: PLoS One. 2024 Oct 28;19(10):e0312775. doi: 10.1371/journal.pone.0312775 (PMC11515975; doi:10.1371/journal.pone.0312775)

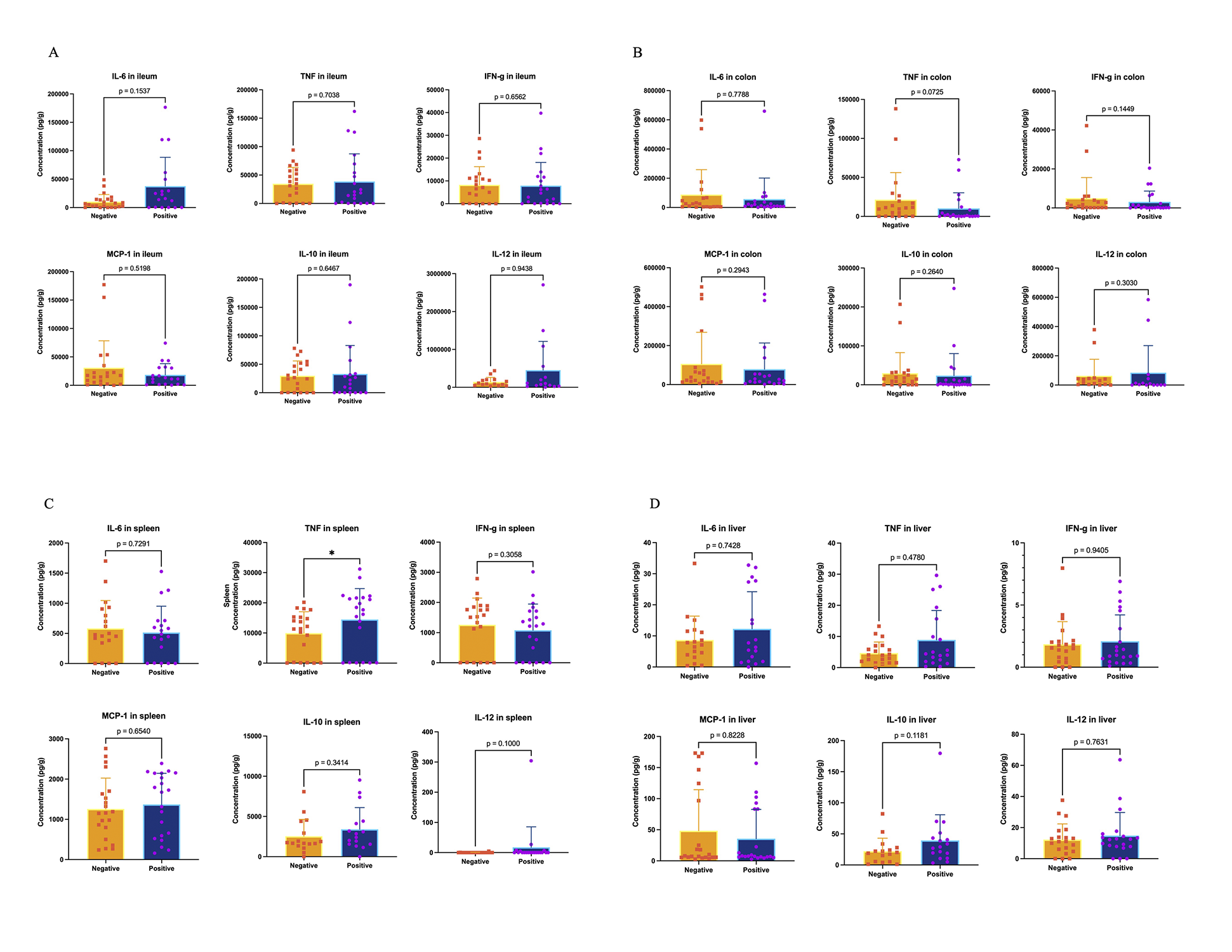

Supplement: S3 Fig — Inflammatory cytokine concentration (pg/g) measured by cytometric bead array assays in the intestine of germ-free mice with protist-negative and protist-associated FMT. Inflammatory cytokines concentrations in the ileum (A), colon (B), spleen (C), and liver (D) of mice with protist-associated and protist-negative FMT are shown as a pool of three experiments and are represented as mean (SD) and differences were tested with Mann–Whitney U-test. p = probability value. (TIF) [file pone.0312775.s003.tif]

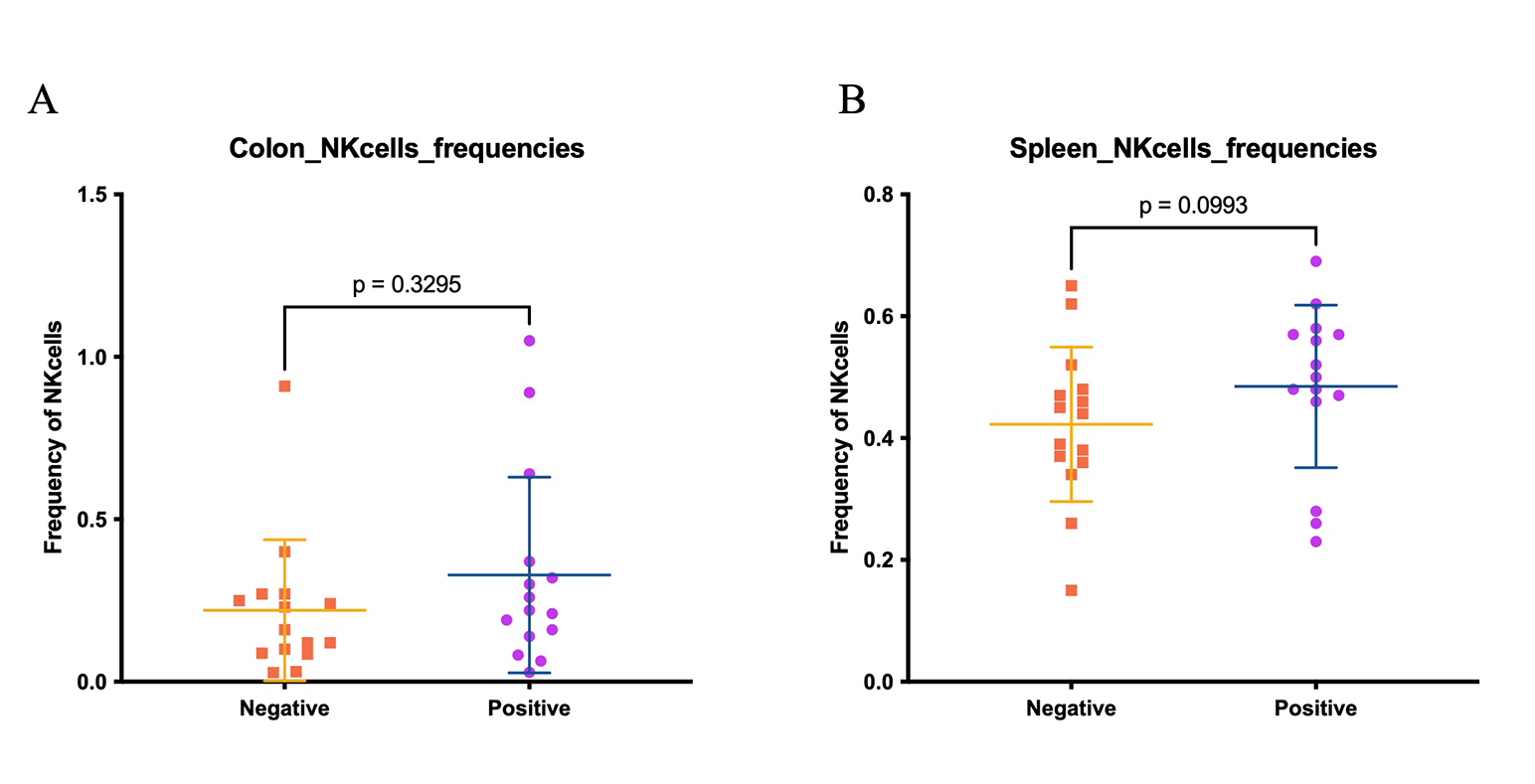

Supplement: S4 Fig — Flow cytometry frequencies (%) of CD3+ CD49b + NK cells in the colon (A) and the spleen (B) of germ-free mice with protist-negative and protist-associated FMT. Results are a pool of three experiments and are represented as mean (SD) and differences were tested with the Mann–Whitney U-test. p = probability value. (TIF) [file pone.0312775.s004.tif]
