# Supplementary material for: Fecal microbiota transplantation from protozoa-exposed donors downregulates immune response in a germ-free mouse model, its role in immune response and physiology of the intestine
Source: PLoS One. 2024 Oct 28;19(10):e0312775. doi: 10.1371/journal.pone.0312775 (PMC11515975; doi:10.1371/journal.pone.0312775)
Supplement: S1 Fig — (PDF) [file pone.0312775.s001.pdf]

**S1. Data and percentages of parasitic protists in the group of volunteers studied.**

PARASITE GROUP

PROTIST PARASITES

| ID | Blastocystis hominis | Entamoeba coli | Entamoeba histolyt | Endolimax nana | Iodamoeba bütschlii | Chilomastix mesnili |
|---|---|---|---|---|---|---|
| P1 | 1 | 1 | 0 | 0 | 0 | 0 |
| P2 | 1 | 0 | 0 | 1 | 1 | 0 |
| P3 | 1 | 1 | 1 | 0 | 0 | 0 |
| P4 | 1 | 1 | 0 | 1 | 0 | 0 |
| P5 | 1 | 0 | 0 | 1 | 0 | 0 |
| P6 | 1 | 1 | 0 | 0 | 1 | 1 |
| P7 | 1 | 1 | 0 | 0 | 0 | 0 |

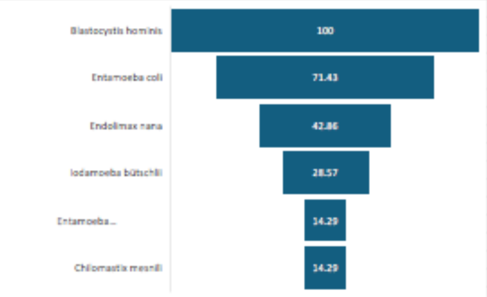


**Percentage of presence of protists in the parasitized group (n=7)**

SOCIODEMOGRAPHIC CHARACTERISTICS OF MOTHERS

| GROUP | ID | AGE (YEARS) | TREATMENT WI | TREATMENT V | GASTROINTESTINAL SYMPTOMS |
|---|---|---|---|---|---|
| PARASITE | P1 | 18 | NOT | NOT | NOT |
| PARASITE | P2 | 31 | NOT | NOT | NOT |
| PARASITE | P3 | 26 | NOT | NOT | NOT |
| PARASITE | P4 | 28 | NOT | NOT | NOT |
| NON PARASIT | NP1 | 34 | NOT | NOT | NOT |
| NON PARASIT | NP2 | 34 | NOT | NOT | NOT |
| NON PARASIT | NP3 | 33 | NOT | NOT | NOT |
| NON PARASIT | NP4 | 30 | NOT | NOT | NOT |

CHARACTERISTICS OF CHILD

| GROUP | ID | SEX | AGE (MONTHS) | TYPE OF BIRT | TYPE OF BREASTF | TREATMENT WIT | TREATMENT WI | GASTROINTESTINAL SYMPTOMS |
|---|---|---|---|---|---|---|---|---|
| PARASITE | P5 | FEMALE | 20 | VAGINAL BIRT | BREASTFEEDING | NOT | NOT | NOT |
| PARASITE | P6 | FEMALE | 22 | VAGINAL BIRT | BREASTFEEDING | NOT | NOT | NOT |
| PARASITE | P7 | FEMALE | 23 | VAGINAL BIRT | BREASTFEEDING | NOT | NOT | NOT |
| NON PARASIT | NP5 | FEMALE | 9 | VAGINAL BIRT | BREASTFEEDING | NOT | NOT | NOT |
| NON PARASIT | NP6 | FEMALE | 15 | VAGINAL BIRT | BREASTFEEDING | NOT | NOT | NOT |
| NON PARASIT | NP7 | FEMALE | 12 | VAGINAL BIRT | BREASTFEEDING | NOT | NOT | NOT |
