# Supplementary material for: Fecal microbiota transplantation from protozoa-exposed donors downregulates immune response in a germ-free mouse model, its role in immune response and physiology of the intestine
Source: PLoS One. 2024 Oct 28;19(10):e0312775. doi: 10.1371/journal.pone.0312775 (PMC11515975; doi:10.1371/journal.pone.0312775)
Supplement: Attachment — Submitted filename: Response to reviewers.pdf [file pone.0312775.s006.pdf]

UNIVERSIDAD NACIONAL AUTONOMA DE MEXICO

RESPONSE TO REVIEWERS (PONE-D-24-07909R1)

Reviewer #2: Fecal microbiota transplantation from protozoa-exposed donors downregulates immune response in a germ-free mouse model, its role in immune response and physiology of the intestine.

Questions

l. 429 pl don't write spp in cursive

Answer: We removed the italics (now in L436).

2. 485, 486 please don't write phylum and families in cursive

Answer: We removed italics in phylum and families (now in L492 and 493).

3. References

Your refs are still affected with many irregularities and citation style mistakes

Answer: We have reviewed and corrected every reference, considering the parameters suggested by PLOS ONE.

UNAM La Universidad de la Nación

Dr. Balmis No. 148, Colonia Doctores, Delegación Cuauhtémoc, Ciudad de México, México, C.P. 06725
Tel. 55 56 23 26 71
